# Supplementary material for: Physicochemical Characterizations, Digestibility, and Lipolysis Inhibitory Effects of Highland Barley Resistant Starches Prepared by Physical and Enzymatic Methods
Source: Molecules. 2023 Jan 20;28(3):1065. doi: 10.3390/molecules28031065 (PMC9920265; doi:10.3390/molecules28031065)
Supplement: Supplementary file 1 [file molecules-28-01065-s001.zip › molecules-2136810-supplementary.pdf]

Supplemental Materials

Figure legends

Figure S1. The morphological property of HBS at the magnifications of 500.0 × (A), 2000.0 × (B), and 5000.0 × (C).

Figure S2. FT-IR spectrum (A) and XRD pattern (B) of HBS.

A

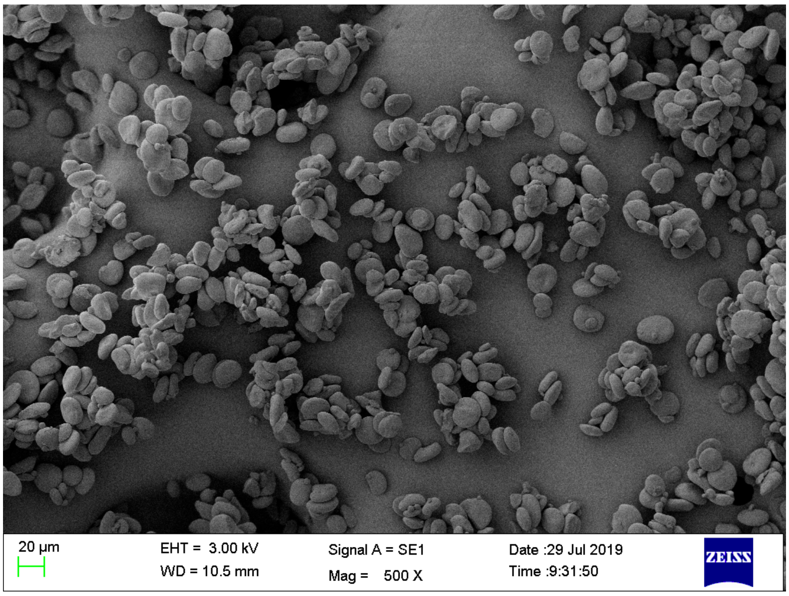

20 µm
EHT = 3.00 kV
WD = 10.5 mm
Signal A = SE1
Mag = 500 X
Date :29 Jul 2019
Time :9:31:50
ZEISS

B

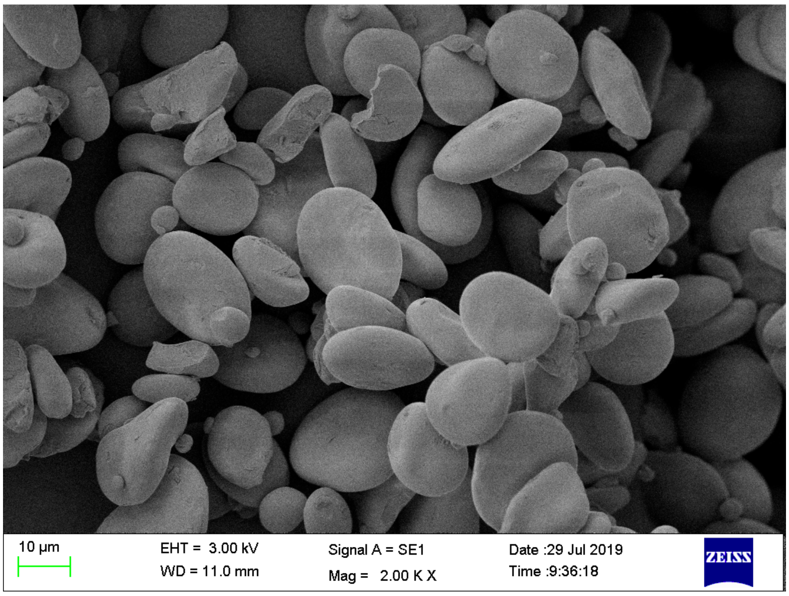

10 µm
EHT = 3.00 kV
WD = 11.0 mm
Signal A = SE1
Mag = 2.00 K X
Date :29 Jul 2019
Time :9:36:18
ZEISS

C

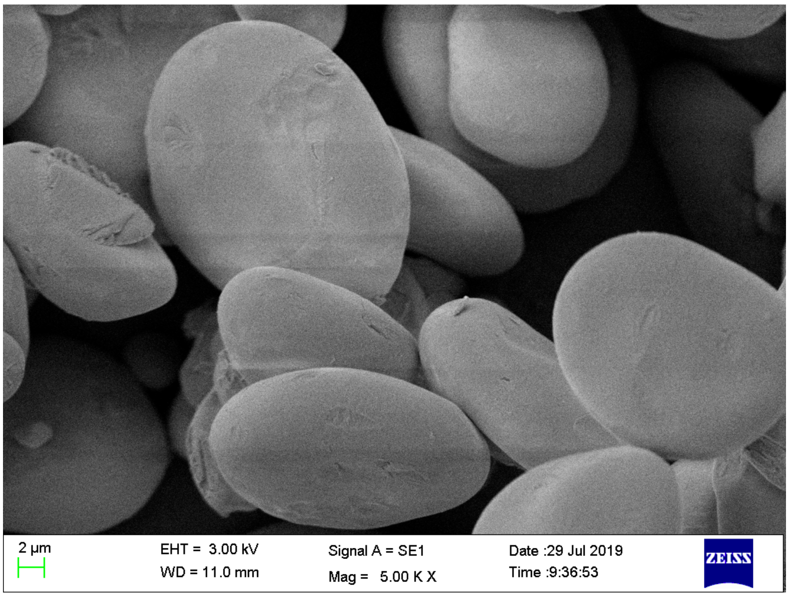

Figure S1: The morphological property of HBS at the magnifications of 500.0× (**A**), 2000.0× (**B**), and 5000.0× (**C**)

A

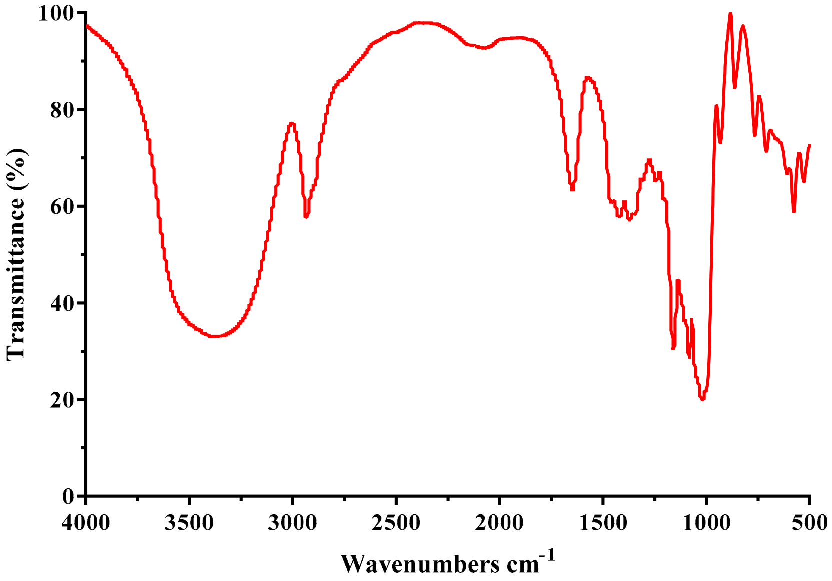

B

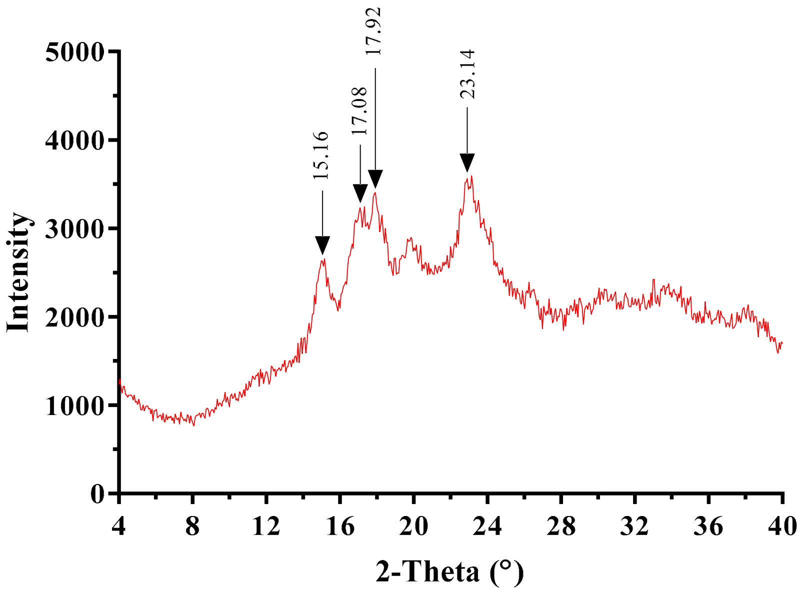

Figure S2: FT-IR spectrum (**A**) and XRD pattern (**B**) of HBS

Tables

Table S1 Physicochemical characterization of HBS.

Samples	HBS
Total starch content (%)	96.82 ± 0.24
Amylose content (%)	32.98 ± 0.82
Protein content (%)	nd [b]
Water-binding capacity (g/g)	137.50 ± 2.12
Solubility (%)	14.75 ± 2.47
Swelling power (g/g)	11.57 ± 0.68
Relative crystallinity (%)	18.03 ± 0.51

[a] % is expressed as the weight percent. [b] nd means no data detected.

The data were presented as means ± SD (n = 3).
